# Adaptation to poststroke visual field loss: A systematic review

**DOI:** 10.1002/brb3.1041

**Published:** 2018-07-13

**Authors:** Claire Howard, Fiona J. Rowe

**Affiliations:** ^1^ Department of Health Services Research University of Liverpool Liverpool UK; ^2^ Department of Orthoptics Salford Royal NHS Foundation Trust Manchester UK

**Keywords:** adaptation, hemianopia, intervention, rehabilitation, stroke, visual field

## Abstract

**Aim:**

To provide a systematic overview of the factors that influence how a person adapts to visual field loss following stroke.

**Method:**

A systematic review was undertaken (data search period 1861–2016) inclusive of systematic reviews, randomized controlled trials, controlled trials, cohort studies, observational studies, and case controlled studies. Studies including adult subjects with hemifield visual field loss, which occured as a direct consequence of stroke, were included. Search terms included a range of MESH terms as well as alternative terms relating to stroke, visual field loss, visual functions, visual perception, and adaptation. Articles were selected by two authors independently, and data were extracted by one author, being verified by the second. All included articles were assessed for risk of bias and quality using checklists appropriate to the study design.

**Results:**

Forty‐seven articles (2,900 participants) were included in the overall review, categorized into two sections. Section one included seventeen studies where the reviewers were able to identify a factor they considered as likely to be important for the process of adaptation to poststroke visual field loss. Section two included thirty studies detailing interventions for visual field loss that the reviewers deemed likely to have an influence on the adaptation process. There were no studies identified which specifically investigated and summarized the factors that influence how a person adapts to visual field loss following stroke.

**Conclusion:**

There is a substantial amount of evidence that patients can be supported to compensate and adapt to visual field loss following stroke using a range of strategies and methods. However, this systematic review highlights the fact that many unanswered questions in the area of adaptation to visual field loss remain. Further research is required on strategies and methods to improve adaptation to aid clinicians in supporting these patients along their rehabilitation journey.

## 1 BACKGROUND

Visual impairment is a common finding after stroke with a recently reported point prevalence of any type of poststroke visual impairment at 72% and incidence of 60% (Rowe, Hepworth, Hanna, & Howard, 2016). Visual impairment may include impaired central vision, impaired peripheral vision (visual field loss), eye movement disorders, and visual perception disorders including visual inattention. Reported prevalence of visual field defects following stroke varies widely and, if present, can have negative implications on quality of life and activities of daily living. Hemianopic visual field defects are associated with a reduced prognosis for successful rehabilitation (Patel, Duncan, Lai, & Studenski, 2000; Han, Law‐Gibson, & Reding, 2002), especially when combined with visual inattention (Cassidy, Bruce, Lewis, & Gray, 1999; Jehkonen et al., 2000). In addition, the extent of visual field loss will impact on the functional symptoms a patient experiences, hence, influencing the adaption process. For example, a patient with macular splitting hemianopia will experience more difficulty with reading tasks than those without this clinical sign (Trauzettel‐Klosinski & Reinhard, 1998). Patients with hemianopic field defects cannot process images in the same way as those with a full visual field. They demonstrate numerous visual refixations and inaccurate saccades which result in impaired scanning, longer search times, and the visual omission of relevant objects (Zihl, 1995a). Visual inattention, otherwise known as visual neglect, can coexist with visual field loss, particularly in strokes located on the right side of the brain (Gottlieb & Miesner, 2004). If field loss is combined with visual inattention, a person typically does not automatically scan or track to the affected side, making adaptation more problematic and less likely to occur.

Treatment for visual field loss is inconsistent and not commonplace, even in stroke units where orthoptic services are provided. There are three main approaches to rehabilitation of visual impairment: adaptation/compensatory, substitution, or restitution as discussed in a 2011 Cochrane review (Pollock et al., 2011). This review concluded that compensatory training was a more favorable option. Such treatment may potentially increase speed of adaptation to the visual loss, but more research is needed in this area. Visual search training usually involves patients practicing identifying objects in their hemianopic and intact hemifields, improving their detection performance over a period of time. There is accumulating evidence that patients can improve their scanning performance with visual search training; however, it is unclear to what extent this training is transferable to everyday life skills, such as obstacle avoidance and increased hazard perception.

In real‐life settings, some people adapt remarkably well to their visual field loss and within weeks of their stroke can read easily, negotiate new surroundings, and appear to have little detriment to their everyday activities, despite having no recovery of their visual field loss. A further group of people appear to be more affected by this loss of vision, struggling with everyday tasks such as reading, mobility, and location of objects around them. The authors have an interest in this specific area as it has been noticed in the clinical setting that there is a wide variation in the way people adapt to their visual field loss. We do not fully understand why some people adapt at a different rate to others. Those who adapt well have a noticeably improved quality of life over those who do not. If we can understand this process in more depth, this allows the potential for clinicians to influence this change in behavior and better support the patients’ adaptation processes. This review aims to investigate current knowledge into the mechanism of adaptation to visual field loss, the factors that influence how a person adapts to visual field loss and the interventions that are available to aid the adaptation process specifically.

We aim to use the systematic review as a starting point for a clinical study to explore the factors that influence the adaptation process in more detail. The findings of the review and clinical study together will be related back to clinical practice, allowing clinicians to target interventions effectively to insure people adapt as quickly and efficiently as possible to visual field loss following a stroke. This review differs from others in the related topic area due to its specific focus on adaptation and the interventions that focus on assisting this process. This is not a full review of the interventions for visual field loss as this has been covered elsewhere (Pollock et al., 2011; Hanna & Rowe, 2017). Similarly, the review will not include restorative rehabilitation or recovery of visual field as this is outside the review objectives.

## 2 METHODS

We conducted a full systematic review of the literature dating from the start of recorded databases for each information source to April 2016, aiming to collect all evidence relating to adaptation to poststroke visual field loss. A detailed protocol was developed prior to the review and registered with PROSPERO (Shamseer et al., 2015).

By the term adaptation, we mean the process whereby people evolve and change behaviors, despite no change in their circumstances, in this instance, an unchanged defect in their visual field. This is different to recovery of visual field, whereby there is a physical change to the area of peripheral vision. We therefore define adaptation in this context to be a persons’ behavioral and practical responses to the visual field loss over time. Adaptation may be a fully conscious reaction such as a person making attempts to move their head more frequently or increase their scanning eye movements or could indeed be factors out of conscious control such as a person's previous visual scanning experiences. This review does not specifically include the process of coping, or a person's emotional response to their visual field deficit. Coping is defined as a person's ability to effectively deal with something difficult, to minimize stress. Coping tends to be a short‐term strategy that is prompted by a lack of alternatives, whereas adaptation involves more sustained planning and focuses on finding alternative ways of handling a task. The terms “adaptation” and “coping” are often used interchangeably, but for the context of this review, the focus is adaptation, making changes to deal with the situation, as oppose to coping or accepting things the way they are.

In general, people adapt to change by forming new expectations that lead to an ability to deal with the new conditions. To adapt to a change in visual status, a person needs to be able to accept the situation and then deal with the implications of this as well as make physical changes and develop strategies to allow them to adapt.

### 2.1 Inclusion criteria

#### 2.1.1 Types of studies

The following types of studies were included: systematic reviews, randomized controlled trials, controlled trials, prospective and retrospective cohort studies, observational studies, and case controlled studies. Case reports, editorials, and letters were excluded. All languages were included, and translations obtained when necessary.

#### 2.1.2 Participants

We included studies reporting on subjects over the age of 18 years only, as children are likely to have different adaptation mechanisms. Studies including subjects with hemifield visual field loss of any severity, which occured as a direct consequence of stroke, were included. Studies reporting on mixed populations were only included if 50% or more of subjects had a diagnosis of stroke and data were available within this category.

#### 2.1.3 Information sources and search strategy

We utilized systematic strategies to search key electronic databases and contacted known experts in the field. We used a range of search strategies as outlined below:

1 We searched the following electronic bibliographic databases:
  - Cochrane Stroke Group Trials Register
  - The Cochrane Eyes and Vision Group Trials Register
  - The Cochrane Central Register of Controlled Trials (CENTRAL) (*The Cochrane Library*, September 2015);
  - MEDLINE (1950 to April 2016);
  - EMBASE (1980 to April 2016);
  - CINAHL (1982 to April 2016);
  - AMED (1985 to April 2016);
  - PsycINFO (1967 April 2016);
  - Dissertations & Theses (PQDT) database (1861 to April 2016);
  - British Nursing Index (1985 to April 2016);
  - PsycBITE (Psychological Database for Brain Impairment Treatment Efficacy, http://www.psycbite.com).
2 The following registers of ongoing trials were searched:
  - ClinicalTrials.gov (http://clinicaltrials.gov/);
  - Current Controlled Trials (http://www.controlledtrials.com);
  - Trials Central (http://www.trialscentral.org);
  - Health Service Research Projects in Progress
  - (http://www.cf.nlm.nih.gov/hsr_project/home_proj.cfm);
  - National Eye Institute Clinical Studies Database (http://clinicalstudies.info.nih.gov/cgi/protinstitute.cgi?NEI.0.html)
3 Hand searching of the following journals was performed to insure full inclusion of relevant studies:
  - British and Irish Orthoptic Journal
  - Australian Orthoptic Journal
  - Proceedings of the European Strabismological Association (ESA)
  - International Strabismological Association (ISA)
  - International Orthoptic Association (IOA) (http://pcwww.liv.ac.uk/~rowef/index_files/Page646.htm)
  - Proceedings of Association for Research in Vision and Ophthalmology (http://www.arvo.org).
4 Reference lists of included articles were hand searched for relevant studies.
5 Experts in the post stroke field of visual field loss were contacted where relevant.

#### 2.1.4 Search terms

Search terms (Table 1) included a range of MESH terms as well as alternative terms relating to stroke, visual field loss, visual functions, visual perception, and adaptation. Due to the specific target area for this review, it was necessary to include search terms for factors that have the potential to influence the adaptation process. These search terms were identified and discussed by a group of stroke survivors who themselves had personal experience of adapting to visual field loss following stroke. The authors were aware that using the term “adaptation” alone would elicit few results, so search terms were included such as driving, reading, saccades, hazard perception, and visual tracking, to encompass the factors considered important for the adaptation process.

**Table 1:** Search terms

Cerebrovascular disorders/ Brain ischemia/ Intracranial Arterial Disease Intracranial Arteriovenous Malformations/ Intracranial Embolism and Thrombosis/ Intracranial Hemorrhage Stroke/	Hemianopsia/ Visual Fields/ Psychological adaptation/ Eye/ Eye Disease/ Visually Impaired Persons/ Vision Disorders/ Blindness/ Vision, Binocular/ Vision, Monocular/ Visual Acuity/ Vision, Low/ Visual Perception/ Automobile driving/ Reading/ Rehabilitation/ Motion perception/ Smooth pursuits Saccades Depth perception Hazard perception Visual tracking Eccentric viewing
OR	OR
AND	

#### 2.1.5 Selection process

The titles and abstracts identified from the search were independently screened by the two authors (CH, FR) through each phase of the review (screening, eligibility, and inclusion) using the prestated inclusion criteria. Where further information was required for this process, the full paper was obtained and the selection criteria applied. A subsequent review of the full papers was undertaken to determine which studies should be included (CH, FR). In the case of disagreement between authors for inclusion, an option was available to seek the opinion of a third reviewer, however, this option was not required in practice as no disagreements occurred.

#### 2.1.6 Data extraction for included studies

A predesigned form was used for the data extraction process. The data extraction form encompassed all the factors identified by stroke survivors as having potential importance for the adaptation process: extent of visual field loss; site of brain lesion; age; gender; ethnicity; handedness; cognition; anxiety levels; social deprivation; preexisting ocular conditions; general signs and symptoms as well as ocular signs and symptoms. Data were extracted by one reviewer (CH) and verified for completeness and accuracy by another (FR).

#### 2.1.7 Quality assessment

One reviewer (CH) reviewed the quality of included studies using the following four checklists; this was subsequently verified by the second reviewer (FR). The term “quality” refers to: “the degree to which a study employs measures to minimize bias and errors in its design, conduct, and analysis” (Khan, Kunz, Kleijnen, & Antes, 2003).

1. CONSORT (Consolidated Standards of Reporting Trials)—for evaluation of the quality of evidence in randomized control and control trials. An adapted version of the CONSORT statement was used (Moher et al., 2010).
2. STROBE (Strengthening the Reporting of Observational Studies in Epidemiology)—an adapted version of the STROBE statement was used to assess the quality of cohort, control, and cross‐sectional studies (von Elm et al., 2007). It is important to note that STROBE measures the reporting quality of the completeness with which a study is presented and the resultant score is not a measure of methodological quality (da Costa, Cevallos, Altman, Rutjes, & Egger, 2011).
3. PRISMA (Preferred Reporting Items for Systematic Reviews)—an adapted version of the PRISMA statement was used to assess evidence in review articles, including Cochrane reviews (Moher, Liberati, Tetzlaff, & Altman, 2009).
4. GRACE (Good ReseArch for Comparative Effectiveness)—an adapted version of the GRACE checklist was used for observational studies. Although the approaches to scoring using this checklist have not been formalized, it has been suggested that if a paper addresses most of the items on the checklist, it can be deemed a reliable source (Dreyer, Velentgas, Westrich, & Dubois, 2014).

Checklists were adapted to insure they only included information considered important to appraise quality of the included studies. Checklist items excluded were not considered by the reviewers as relevant to the appraisal process; for example, title, background, funding, and setting.

## 3 RESULTS

**Figure 1:**
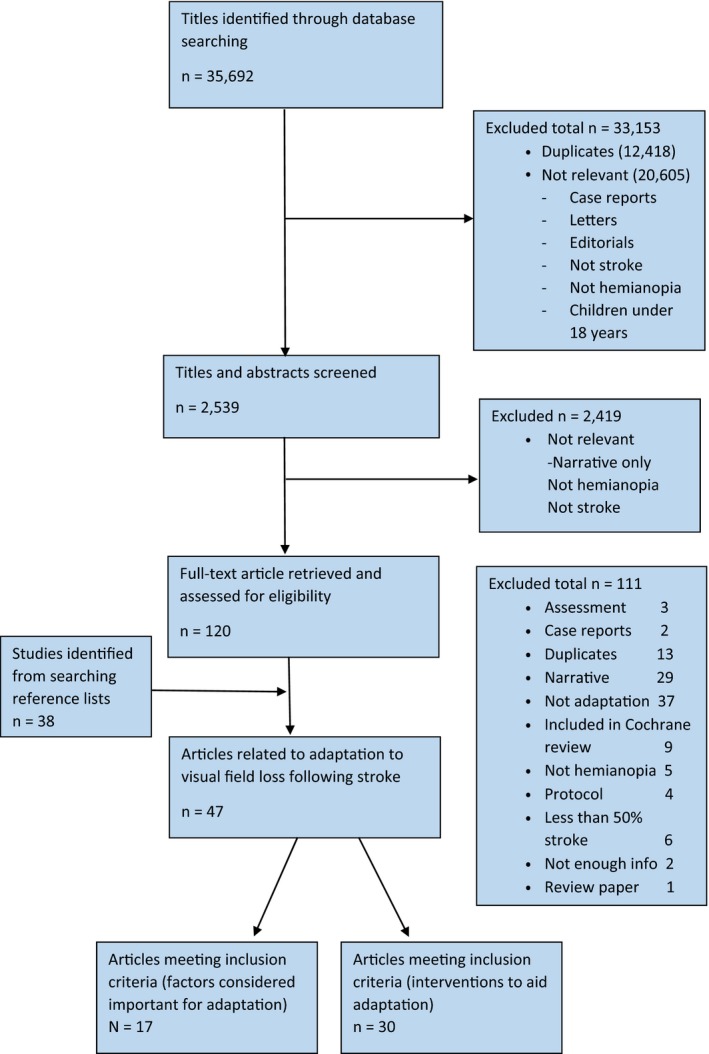
Overview of search results

Results of the search are outlined in Figure 1. As expected, there were no identified studies which explored the factors that influence how a person adapts to visual field loss following stroke in a precise and systematic manner. In other words, no one article has explored and discussed all of the factors important for the adaptation process over time to answer this question fully. However, there were seventeen articles identified by the reviewers as containing a factor considered likely to be important for the process of adaptation to poststroke visual field loss. These were articles that contained information on the factors considered as potentially important for the adaptation process by the group of stroke survivors themselves. These articles, covering factors such as age, environment, compensation strategies, and awareness of symptoms, are discussed as a group. There were thirty additional studies identified that focused on the interventions for visual field loss that were deemed directly related to the factors above. Only articles that focused on adaptation factors or interventions likely to influence these were included, making this review distinct from other intervention reviews. In summary, a total of 47 articles (2,900 participants) were included in the overall review, divided into two sections for reporting:

- Studies where the reviewers could identify a factor they consider is likely to be important for the process of adaptation to poststroke visual field loss.
- Studies that detailed interventions relating to the above factors.

Due to the variations and diversity across trials, with respect to reporting of outcomes as well as recruitment and selection of subjects, a meta‐analysis of studies was not undertaken. A narrative summary of the data is presented in relation to included studies.

### 3.1 Factors that have the potential to affect adaptation

There were seventeen articles (1,423 participants—with 809 of these having poststroke visual field loss) included in this section of the review. Table 2 summarizes the key data extracted from the studies including the proposed link to adaptation as deemed by the reviewers and stroke survivors. The seventeen articles included studies consisting of one randomized controlled trial, eleven cohort studies, and five prospective observational studies.

**Table 2:** Data extracted from 17 articles deemed significant to adaptation process

Study	Study design	Aim/objective	Sample size (*n*)	Population	Intervention	Intervention duration	Link to adaptation	Results summary
Baier et al. (2015)	Prospective observational study	To analyze the brain regions specifically related to anosognosia for visual field defects.	*n* = 54 41 hemianopia 13 quadrantanopia	Stroke	n/a	n/a	Awareness: lack of symptoms may impact adaptation.	An association between anosognosia for field defects and parts of the lingual gyrus, the cuneus, posterior cingulate and the corpus callosum.
Cassidy et al. (1999)	Cohort study	To determine whether the presence of a visual field defect exacerbates visuospatial neglect.	*n* = 44 20 field defect and neglect 7 neglect only I field defect only 17 control	Stroke	n/a	n/a	Awareness: presence of neglect may impact adaptation.	Patients with field defect and neglect had lower scores on behavioral inattention tests
Celesia et al. (1997)	Prospective observational study	To investigate awareness of visual field loss in hemianopia	*n* = 32	Stroke	n/a	n/a	Awareness: lack of symptoms may impact adaptation.	Hemianopic anosognosia is most often related to failure of discovery of the deficit or occasionally by severe hemineglect or cognitive impairment.
Hardiess et al. (2010)	Cohort study	Investigate role of eye and head movements as a compensatory strategy in hemianopia and normal controls	*n* = 24 12 hemianopia 12 control	Mixed Stroke *n* = 11 Surgery n = 1	n/a	n/a	Compensation strategy: eye and head movements	Hemianopic patients showed increased gaze movement activity.
Kasneci et al. (2014)	Cohort study	Assess impact of hemianopia on a supermarket search task.	*n* = 20 10 hemianopia 10 control	Mixed Stroke *n* = 8 Trauma *n* = 1 Surgery *n* = 1	n/a	n/a	Compensation strategies: eye movements/visual search	A considerable number of patients with hemianopia could compensate by shifting their gaze toward the peripheral visual field and the visual field area
Loverro and Reding (1988)	RCT	Assess effect of bed orientation on rehab outcome for patients with hemianopia or visual neglect	*n* = 44 24 hemianopia 20 neglect	Stroke	Bed orientation to ipsilateral or contralateral side of infarct.	Variable: throughout rehab stay	Environment: bed orientation/side of stimulation	Patients with field defects improve equally well irrespective of bed position.
Machner et al. (2009)	Cohort study	Record eye movements of hemianopic patients to explore disorders of visual search	*n* = 18 9 hemianopia 9 control	Stroke	n/a	n/a	Compensation strategies: saccades/visual search	Abnormal visual search in acute hemianopia is related to the brain lesion.
McDonald et al. (2006)	Cohort study	Comparison of reading scanpaths between patients with hemianopic alexia and normal controls.	*n* = 28 18 hemianopia 10 control	Mixed Stroke *n* = 15 Tumor *n* = 2 Brain injury 1	n/a	n/a	Compensation strategies: reading strategies/scanpaths	Patients were able to extract useful visual information from text to aid the planning of reading scanpaths.
Meienberg et al. (1981)	Prospective observational study	Explore compensatory strategies used in hemianopes to find and fixate objects, using infrared oculographic recordings.	*n* = 3	Stroke	n/a	n/a	Compensation strategies: saccades/visual search	Hemianopic patients employed a consistent set of compensatory strategies to find and fixate objects.
Pambakian et al. (2000)	Cohort study	Examine scanpaths of patients with hemianopia while viewing pictures.	*n* = 16 8 hemianopia 8 control	Mixed Stroke *n* = 4 Tumor *n* = 2 AVM n = 2	n/a	n/a	Compensation strategies: saccades/scanpaths	Various features of the scanpaths produced by hemianopes were different from normal subjects.
Papageorgiou et al. (2012)	Cohort study	Identify efficient compensatory gaze patterns applied by patients with hemianopia under virtual reality conditions in a dynamic collision avoidance task.	*n* = 60 30 hemianopia 30 control	Mixed Stroke *n* = 24 Trauma *n* = 4 Surgery *n* = 2	n/a	n/a	Compensation strategies: gaze patterns/visual scanning	Patients with hemianopia who adapt successfully display distinct gaze patterns, with increased eye and head movements.
Rowe et al. (2013)	Prospective multicentre case cohort study	Profile site of stroke, type and extent of field loss, treatment, and outcome.	*n* = 915 479 with field loss 151 with field loss as only complaint	Stroke	Compensatory: typoscope, exercises, advice Substitutive: peli prisms, prisms	Variable—standard practice	Awareness: lack of symptoms may impact adaptation.	Stroke survivors with visual field loss need assessment to define type and extent of loss, diagnose coexistent impairments and offer targeted treatments.
Schuett and Zihl (2013)	Prospective observational study	Determine the effect of age in patients with hemianopia receiving compensatory oculomotor treatment for their reading and visual exploration impairments	*n* = 38	Mixed Stroke *n* = 33 Trauma *n* = 2 Tumor *n* = 3	Compensatory: visual exploration training, reading, and training	Training sessions of 45 min each. Patients required on average nine sessions of training within 2–3 weeks.	Effect of age Compensation strategies: reading and visual exploration	Older patients achieve the same treatment induced improvements as younger patients.
Tant et al. (2002)	Cohort study	To investigate scanning performance in simulated and real hemianopia. Also to observe age‐related processes in compensating for hemianopia	*n* = 45 16 healthy subjects (simulated hemianopia) 29 hemianopia	Mixed Stroke *n* = 27 Tumor *n* = 2	n/a	n/a	Effect of age Compensation strategies : scanning behavior	There were clear parallels between simulated and real hemianopia suggesting hemainopic scanning behaviour is primarily visually elicited.
Taylor et al. (2012)	Prospective observational study	To investigate the effects of a treatment program on head and shoulder movement for people with visual field defects.	*n* = 2	Stroke	Compensatory: training head movements in isolation to shoulder movement	30 min sessions twice weekly for 4 weeks	Compensation strategies: encouraging head movements	Head and shoulder movements change following a field defect after stroke.
Wood et al. (2011)	Cohort study	Compare eye and head movements, lane keeping and vehicle control of drivers with hemianopia and quadrantanopia with controls.	*n* = 60 22 hemianopia 8 quadrant'opia 30 control	Mixed Stroke *n* = 18 AVM *n* = 2 congenital *n* = 2 Trauma *n* = 5 Tumor *n* = 3	n/a	n/a	Compensation strategies: head and eye movements	People with visual field defects rated a safe to drive compensated by making more head movements into their blind field.
Zangemeister and Oechsner (1996)	Cohort study	Observe short‐term adaptation to hemainopia by analyzing visual search, reading, and scanpath eye movements.	*n* = 20 10 hemianopia 10 control	Stroke	n/a	n/a	Compensation strategies: scanning behavior	Study demonstrated short term adaptation as a result of short term training in hemianopic patients.

#### 3.1.1 Quality assessment

The quality of evidence was assessed for each of the 17 articles included in this section (Supporting Information Tables [Link], [Link], [Link]). Evidence was considered as good quality if the article scored 75% or greater on the relevant checklist. In summary, no articles scored 100% for quality of evidence in this section, in the opinion of the reviewers. Twelve articles scored between 75% and 99% and, therefore, deemed as good quality evidence. Three scored between 50% and 74% on the relevant quality checklists, and two articles failed to reach 50%, achieving 48% (Loverro & Reding, 1988) and 44% (Taylor, Poland, & Stephenson, 2012), respectively. All articles were included in the review with quality of evidence taken into consideration in the discussion.

The factors extracted as likely to be important for the adaptation process covered five different areas: compensation strategies used by an individual; a person's awareness of their hemianopia; presence of inattention; effect of age; and environment surrounding a person during the poststroke period. The articles identified in each section will be discussed individually. There are likely to be a number of further factors important for the adaptation process which will need to be explored in more detail by further research, but we did not elicit any results within the remit of this review, relating specifically to adaptation. These factors include previous visual experiences, occupation of the patient prestroke, site of the brain lesion and perhaps most importantly, the extent of visual field loss. It is feasible to suggest that someone with a more extensive visual field loss will adapt in a different manner to someone with a field loss of a lesser extent. No articles reported on the direct relationship between extent of visual field loss and/or the presence of macular sparing and their importance in the process of adaptation. This is a noted limitation of this review in that the authors cannot comment on an association between extent of field loss and its importance in the adaptation process; this factor warrants further exploration. Several of the included articles detailed the extent of visual field loss in their patients (Hardiess, Papageorgiou, Schiefer, & Mallot, 2010; Bergsma, Leenders, Verster, van der Wildt, & van den Berg, 2011; Kasneci et al., 2014), but this extent of loss was not related to adaptation in any way. One study by Trauzettel‐Klosinski and Reinhard (1998) reported that the presence or absence of macular sparing influenced factors such as fixation behavior and reading performance. They found that the lesser the extent of macular sparing, the less stable the fixation. This finding is likely to influence the process of adaptation, in particular, when considering adaptive strategies such as eccentric fixation and predictive saccadic eye movements.

#### 3.1.2 Compensation strategies

Ten of the included studies discuss the impact of compensation strategies for poststroke visual field loss including use of eye movements, visual search, head movements, spatial localization, and scanning behavior. Compensation strategies may include the use of head and shoulder movements to aid tasks such as searching, obstacle avoidance and hazard perception, scanning the environment, and/or saccadic eye movements/eccentric fixation for the purpose of improving close tasks such as reading.

Reading/saccadic adaptation—whereas visual acuity testing demands recognition of one optotype at a time, reading demands a more complex simultaneous overview of a group of letters. Patients with hemianopic visual field defects develop compensation strategies to aid reading ability using eccentric fixation and scanning eye movements. Eccentric fixation may help some patients with macular splitting and training in the strategies required for reading can help a patient to adapt to their loss. Eccentric fixation shifts the visual field deficit toward the affected side, creating a small useful visual field area along the vertical meridian. This adaptive strategy benefits a persons’ ability to adapt, particularly for reading tasks (Trauzettel‐Klosinski & Reinhard, 1998). A study by Meienberg, Zangemeister, Rosenberg, Hoyt, and Stark (1981) reported patients as developing compensatory search strategies to overcome difficulties with locating objects. To fixate targets in the seeing hemifield, subjects in this study were shown to undershoot the target to prevent losing it in the blind field, then hold it off the fovea on the seeing side of the macula. This is considered a useful strategy for improving reading ability in this group of patients. Meienberg et al. (1981) also discussed the difference between short‐ and long‐term adaptation. In the short term, patients with hemianopia develop a staircase strategy to search for a target, whereas in the longer term, they employ a more efficient strategy of one large saccade to overshoot the target. In homonymous hemianopia with macular splitting, severe reading problems result from a loss of half of the reading visual field. This longer term adaptation was further highlighted in a study by Reinhard, Damm, Ivanov, and Trauzettel‐Klosinski (2014) who found that patients with hemianopia showed significantly more dysmetric saccades to the blind side compared to the seeing side. The number of dysmetric saccades, however, did not correlate with duration of hemianopia, indicating insufficient spontaneous long‐term adaptation in the patients.

Although a considerable amount of research has focused on hemianopic reading difficulties or hemianopic alexia and a persons’ ability to compensate for this, we still do not fully understand why some people adapt to this reading difficultly more effectively than others. Patients with hemianopia are reported to employ reading strategies that are inefficient and slower than those with a full visual field.

The severity of the reading problem is also influenced by the side of the defect, in relation to the direction of reading. In left to right readers (as in the English language), a right hemianopia significantly impairs reading as the person cannot see the oncoming groups of letters or words (Trauzettel‐Klosinski & Reinhard, 1998). A left‐sided hemianopia causes problems locating the start of a line of text, meaning they tend to skip lines or restart the same line twice. Those with right‐sided hemianopia show prolonged search durations, prolonged fixation times, reduced amplitudes of saccades to the right, and multiple regression saccades (Machner et al., 2009; Zihl, 1995b). Patients with a right sided visual field loss tend to lose the word they are fixing on, requiring a refixation toward the word in view. This refixation slows their reading time considerably (McDonald, Spitsyna, Shillcock, Wise, & Leff, 2006). How a person compensates for their reading difficulties and uses their visual scanning techniques is likely to impact on the overall process of adaptation, but again, this direct link has not been explored.

Search tasks—When a person experiences a loss of visual field they learn over time to compensate for their visual difficulties by improving the accuracy and speed of eye movements to the defective side. The development of adaptive eye movement strategies over time has been well documented, and the implication of these compensation strategies is that subjects develop a more effective visual search technique, for a variety of tasks such as obstacle avoidance and driving (Hardiess et al., 2010; Meienberg et al., 1981; Machner et al., 2009; McDonald et al., 2006; Pambakian et al., 2000; Papageorgiou, Hardiess, Mallot, & Schiefer, 2012; Zangemeister & Oechsner, 1996; Wood et al., 2011; Roth et al., 2009). This improved visual search is likely to be an important factor in the adaptation process, and this theory has been explored by Roth et al. (2009) in that their study showed explorative saccadic training to improve saccadic behavior, search skills, and scene exploration on the hemianopic side, showing benefits of compensatory exploration training which are transferable to everyday tasks. The interventions targeted by these strategies will be discussed in more detail in the second section of this review. Kasneci et al. (2014) reported on the impact of visual search on a supermarket searching task, to explore the relationship between visual field defects and quality of life. This supermarket search study confirmed a shift of gaze toward the visual field loss in hemianopic patients, providing insight into an everyday task that many people find a struggle when living with this visual impairment.

Some individual studies have focused on one specific aspect of everyday functionality concerning compensation strategies, but no one study has compiled the factors likely to be important for adaptation together into one study. A pilot study by Taylor et al. (2012) provides preliminary information regarding the development of head and shoulder movement strategies as a compensation mechanism following visual field loss. They suggest that head and shoulder movements could be an important factor for the compensation process. This theory needs investigation, with further research warranted in this area.

#### 3.1.3 Awareness of hemianopia/presence of symptoms

Three studies provided information regarding a lack of awareness of hemianopia. The authors of this review feel this has the potential to be an important factor for the adaptation process, as a lack of awareness could affect a persons’ ability to adapt and compensate. In a prospective study of patients with homonymous visual field defects (Celesia, Brigell, & Vaphiades, 1997), 62% of patients were found to have hemianopic anosognosia, defined as the unawareness of visual loss in the homonymous hemifield. Celesia et al. (1997) suggest that this anosognosia is most often related to a failure of discovery of the deficit, occasionally due to severe visual hemineglect, a generalized cognitive impairment or a combination of these factors. A further study of anosognosia for visual field defects reports a lower incidence of 19% of patients failing to recognize their defect (Baier et al., 2015). A multicentre cohort study by Rowe and the VIS group (Rowe et al., 2013) supported this finding and reported 16% of their 479 patients with a visual field loss as not complaining of visual field loss specifically. In this cohort of patients, 10.6% of those with visual field loss were reported as not complaining of any visual symptoms of any type.

#### 3.1.4 Presence of inattention

Although no studies were identified in highlighting inattention as a factor influencing the adaptation process, one paper by Cassidy et al. (1999) reports on the reduced prognosis for patients presenting with inattention in combination with hemianopia. They report on the presence of visual field defects being associated with a more severe form of visuospatial neglect in the first week after stroke, than those without visual field loss. This fact has potential to influence the adaptation process, particularly in the early poststroke stages.

#### 3.1.5 Effect of age

Two studies provide observations around the effect of age on compensation strategies. Older age is generally considered to have an adverse effect on functional outcome following acquired brain injury; therefore, older age is considered likely to be a factor that has potential to influence the adaptation process to poststroke visual field loss. Schuett and Zihl (2013) report findings from their study to determine the effect of age on reading and visual exploration impairments, following compensatory oculomotor treatment. They report that older patients achieve the same post treatment improvements in reading and visual exploration as younger patients, concluding that age does not appear to be a critical factor for functional outcome when focusing on compensatory treatments of visual field defects. These findings suggest that older age is not necessarily associated with a reduced level of adaptation. However, a study by Tant, Cornelissen, Kooijman, and Brouwer (2002) compared scanning performance for healthy subjects on two different occasions, comparing subjects’ own normal performance with their own performance when a hemianopia was simulated. They observed age‐related processes in compensating for the simulated hemianopia. During eye movements recordings, they report a reduced level of compensation to visually elicited disabilities, in the older age ranges. Tant et al. (2002) suggest that disabilities in scanning performance are more pronounced in an older age group, suggesting a possible reason for this as differences in important factors for the compensation process (such as perceptual and intellectual abilities) which tend to decrease with age. There are limitations of this study in that the hemianopic visual field defects assessed were simulated and not true defects caused by brain injury, but the authors of this review feel it warrants a mention as having potential significance for adaptation. Tant et al. compared scanning performance in the simulated hemianopia individuals, the same individuals without the simulated hemianopia, and real hemianopia patients. They reported clear parallels between simulated and real hemianopia, suggesting that hemianopic scanning behavior is elicited by the visual field defect and not by the additional brain impairment. The relationship between age and adaptation requires future exploration if all aspects of the adaptation process are to be considered.

#### 3.1.6 Environment

One study (Loverro & Reding, 1988) detailed the effect of environment, more specifically bed orientation on the outcome for hemianopic patients. Loverro and Reding (1988) found no relationship between bed positioning and rehabilitation outcome in patients with poststroke homonymous hemianopia or visual inattention. In this study, patients with hemianopia or inattention were randomized to have their impaired or unimpaired side directed toward the side of stimulating environment. This article was considered as low quality during the quality assessment process (48%) and the topic of bed positioning and environment is an area that lacks evidence and should be the focus of future research. The authors of this review consider that environment and side of stimulation have the potential to be an important factor in the adaptation to poststroke hemianopia.

### 3.2 Interventions that may influence adaptation

Included in this section were thirty studies (1,477 participants—with 1,411 of these having poststroke visual field loss). This number includes one Cochrane review relating to interventions available for visual field loss following a stroke (Pollock et al., 2011). In view of the rigorous methods employed for Cochrane reviews, the findings have been summarized in this review, followed by a narrative overview of additional articles not included in the Cochrane review. Table 3 summarizes the key data extracted from the included studies and those studies excluded from this review due to inclusion in the Cochrane review (Pollock et al., 2011)—in total nine studies. The thirty included studies consisted of one Cochrane review, six randomized controlled trials, eight cohort studies, eight prospective observational studies, two crossover trials, two noncontrolled trials, two feasibility studies, and one case series.

**Table 3:** Data extracted from 30 articles detailing interventions targeted at adaptation process

Study	Study design	Aim/objective	Sample size (*n*)	Population	Intervention	Intervention duration	Results summary
Aimola et al. (2014)	RCT	Evaluate the efficacy and feasibility of an unsupervised reading and exploration computer training for hemianopia.	*n* = 52 Intervention 28 Control 24	Mixed Ischemic stroke *n* = 39 Hemorrhage *n* = 6 TBI *n* = 6 Tumor *n* = 1 At least 3 months poststroke	Compensatory: computer‐based reading and visual exploration training v sham exploration task	Experimental group: 14 blocks per day Control group: 10 blocks per day One hour sessions for up to 10 weeks	Home based compensatory training for hemianopia can result in objective benefits in searching and reading as well as quality of life.
Bergsma et al. (2011)	Cohort study	To investigate Visual Restorative Function Training (vRFT)‐induced changes in oculaomotor behavior using a driving stimulator	*n* = 12 Hemianopia 6 Controls 6	Stroke	Compensatory: change in oculomotor behavior	65 hr training	vRFT with mandatory eye fixation can result in increased eye movement behavior the defect.
Bolognini et al. (2005)	Cohort study	To verify whether a systematic audio–visual stimulation might induce a long lasting amelioration of visual field disorders	*n* = 8	Chronic visual field loss—cause not stated	Compensatory: audio–visual stimulation of visual field	4 hr daily Duration of nearly 2 weeks	Patients showed improvement of visual detection and visual oculomotor exploration following training.
Bowers et al. (2014)	Double masked randomized crossover trial	Evaluate efficacy of real relative to sham peripheral prism glasses	*n* = 61	Stroke At least 3 months poststroke	Substitutive: 57 ∆ prism placed above and below visual axis v sham 5 ∆ prism.	Each set of prisms worn for 4 weeks. Measured at 6 months.	Real peripheral prism glasses were more helpful for obstacle avoidance when walking than sham glasses, with no difference between horizontal and oblique designs.
de Haan et al. (2015)	RCT	To examine the effects of a compensatory scanning training program using horizontal scanning on mobility‐related activities and participation in daily life	*n* = 54 Results in analysis*n* = 49	Mixed Ischemic stroke *n* = 36 Hemorrhage *n* = 5 TBI *n* = 3 Trauma *n* = 1 AVM 1 Combined 3	Compensatory: InSight‐Hemianopia Compensatory scanning training	15× sessions of 60–90 min each Total 18.5 hr face to face training over 10 weeks.	Horizontal scanning training improved mobility related activities.
Gall and Sabel (2012)	Prospective noncontrolled trial	Examine whether increased visual functioning after VRT coincides with improved reading abilities	*n* = 11	Mixed Infarct *n* = 7 AVM *n* = 1 Haemorrhage *n* = 1 SAH *n* = 1 Encephalitis *n* = 1	Restitutive: VRT	30 min 2× daily 6 days a week Duration 6 months	VRT improved visual fields in parafoveal areas, which are most relevant for reading.
Giorgi et al. (2009)	Cohort study	Evaluate peli prisms as a low vision optical device for hemianopia (extended wearing trial)	*n* = 23	Mixed Stroke *n* = 16 Surgery *n* = 4 TBI *n* = 2 Congenital *n* = 1	Subsitutive: 40∆ prism placed above and below the visual axis	Peli prisms worn for 6 weeks, 3 months and long‐term (duration not specified).	Peripheral prism glasses showed reported benefits to 2/3 of patients in the study.
Hayes et al. (2012)	Case series	Evaluate functional changes following NVT program for poststroke heminaopia	*n* = 13	Stroke Between 2 weeks and 6 months poststroke	Compensatory: NVT	One hour per session, 3× per week for 7 weeks	NVT intervention resulted in functional improvements in mobility post rehabilitation.
Hazelton et al. (2015)	Feasibility study	To explore the use of four different home‐based scanning training interventions for visual field loss	*n* = 12	Stroke At least 6 months poststroke	Compensatory: scanning training (paper‐based Rainbow readers, computer software VISIOcoach, web‐based Happy Neuron and specialized equipment NVT)	Four scanning interventions delivered in randomized order for around 2 weeks.	Home based scanning training is feasible. Key factors in maximising use include levels of cognitive impairment and participant perceptions.
Jacquin‐Courtois et al. (2013)	Prospective observational study	Test the effect and specificity of a compensatory eye movement training therapy	*n* = 7	Mixed Stroke *n* = 5 Tumor *n* = 2 Chronic field loss	Compensatory: Visual search	1× 30 min session	Results show that rapid, compensatory changes can occur in patients with visual field defects.
Jobke et al. (2009) Article taken from cochrane review Pollock et al. (2012)	Randomized, double blinded, crossover study	To compare extrastriate vs conventional VRT in patients with visual field loss	*n* = 21	Mixed Stroke/ ischemia *n* = 10, Cranio‐cerebral injury *n* = 3, Surgery *n* = 3, tumor *n* = 1, meningitis *n* = 1	Restitutive: Extrastriate VRT vs Conventional VRT	Extrastriate 30mins daily for 90 days. Then crossover of conventional VRT for 90 days	Detection performance increased twice as much after extrastriate VRT (4.2%) than after standard VRT (2.4%). NEI‐VFQ did not show any significant changes.
Kasten et al. (1998) Article taken from cochrane review Pollock et al. (2012)	RCT, double blinded	To assess the effect of computer‐based training to treat partial blindness	*n* = 19	Mixed Stroke *n* = 10 Trauma *n* = 4 Other *n* = 5	Restitutive: VRT	1 hr per day, 6 days per week for 6 months Total = 150 hr	In postchiasma patients, VRT led to a significant improvement (29.4%) over baseline in the ability to detect visual stimuli.
Keller and Lefin‐Rank (2010)	RCT	To compare two approaches of blind field exploration in those with recent onset hemianopia and to analyze possible changes in eye movement patterns after training	*n* = 20	Mixed Stroke *n* = 18 Trauma *n* = 1 Tumor *n* = 1	Compensatory: audio–visual stimulation training v visual stimulation training	Both groups received 20 therapy sessions, each session lasting 30mins. Over 3 weeks	Multimodel audiovisual exploration training appears to be more effective than exploration training alone.
Kerkhoff et al. (1992)	Prospective observational study	To determine whether reading speeds and accuracy can be improved with reading training in hemianopic alexia	*n* = 56	Mixed Stroke *n* = 46 Trauma *n* = 8 Tumor *n* = 1 Hypoxia *n* = 1	Compensatory: reading moving text	15–40‐min treatment sessions	The new training procedure can lead to a significant and stable improvement of reading in patients with hemianopic alexia.
Kerkhoff et al. (1994)	Cohort study	To evaluate the efficacy of a systematic training of saccadic eye movements in hemianopic patients	*n* = 22	Stroke	Compensatory: saccadic eye movement training	30 min daily sessions 5 days per week. 25–27 total treatment sessions	Training of compensatory eye movements strategies restores oculomotor functions and improves visual performances.
Lane et al. (2010)	Nonrandomized controlled trial	Explore the efficacy of a visual exploration training	*n* = 42	Mixed Ischemic *n* = 28 Hemorrhage *n* = 10 TBI *n* = 4	Compensatory: Visual exploration training and visual attention training	Exploration training = 40 min sessions over 2–9 weeks Attention training = 30‐min sessions over 2–7 weeks	Both the exploration training and the attention training led to significant improvements in most of the visual tasks.
Lévy‐Bencheton et al. (2016)	Cohort study	To evaluate and compare the effect of an original hemianopia rehab method based on a single 15 min voluntary antisaccades task	*n* = 14	Stroke At least 6 months poststroke	Compensatory: adaptation of anti‐saccades	3 training sessions, separated by 4–5 weeks Each session 15–20 min	Anti saccade training resulted in significant functional improvements in the patient group.
Mannan et al. (2010)	Prospective observational study	To characterize changes in eye movements resulting from training	*n* = 29	Mixed Infarct *n* = 22, hemorrhage *n* = 6, surgery *n* = 1, tumor *n* = 2 At least 3 months poststroke	Compensatory: Visual search training	20 × 40 min sessions for 1 month	Results suggest that visual training facilitates the development of specific compensatory eye movement strategies.
Marshall et al. (2010)	Longitudinal cohort	To determine whether visual field expansion occurs with VRT	*n* = 7	Stroke	Restitutive: VRT	20–30 min 2× daily, 6 days a week For 3 months	There was an average improvement in stimulus detection rate by 12.5%.
Mazer et al. (2003)	RCT	To compare driving performance after useful field of view retraining (UFOV) compared to traditional visuoperceptual retraining	*n* = 84	Stroke	Compensatory UFOV v commercially available computer‐based visuoperceptual retraining (control)	Both groups received 20 sessions (each session30–60 min long) 2–4 sessions per week	Rehabilitation targeting visual attention skill was not significantly more beneficial than traditional percpetual training for on road driving evaluation.
Nelles et al. (2001)	Prospective observational study	Investigate whether training eye movements would induce change in the neural activity of cortical visual areas	*n* = 45 Hemianopia 21 Controls 24	Stroke Infarct *n* = 16 Hemorrhage *n* = 5	Compensatory: Eyes fixating v exploratory eye movements	30 min per session, 2× daily For 4 weeks	Training improved detection of and reaction to visual stimuli without restitution of the visual field defect.
Nelles et al. (2010)	Prospective observational study	Using fMRI to study the training effects of eye movement training on cortical representation of visual hemifields	*n* = 8	Ischemic stroke	Compensatory: Eye movement training	30 min session 1× daily for 4 weeks	Eye movement training induced altered brain activation in the unaffected extrastriate cortex.
Ong et al. (2012)	Longitudinal cohort study	To determine whether Eye‐search web‐based hemifield search training improves patients’ search time and “real world” outcomes.	*n* = 33	Stroke Infarct *n* = 14 Hemorrhage *n* = 3, AVM *n* = 1 unknown *n* = 15	Compensatory: OKN therapy	20 min of therapy per day suggested. Patients prompted to test reading speed after 5 hr of therapy	Read‐Right therapy produced significant improvements in text reading speeds at all time points with a clear dose effect: 10% at 5 h, 20%at 10 h, 39%at 15 h and 46%at 20 h.
Ong et al. (2015)	Prospective observational study	Evaluate efficiency of eye movements following visual search training	*n* = 78	Mixed Stroke *n* = 60 Tumor *n* = 6 TBI *n* = 2 Other *n* = 10	Compensatory: Scanning exercises	11 days of therapy (length of each session not specified)	After therapy, search times into the impaired field improved by an average of 24%.
Pambakian et al. (2004)	Prospective observational study	Examine whether directing attention to ARV using a visuospatial cue also increases long‐term neural plasticity	*n* = 31 29 completed	Mixed Infarct *n* = 22 Hemorrhage *n* = 6 Surgery *n* = 1 Tumor *n* = 2 At least 3 months poststroke.	Compensatory: Visual search training	20× 40 min sessions Sessions in 1 month	Patients can improve visual search with practice.
Passamonti et al. (2009)	Prospective observational study	To study the effects of multisensory training on occulomotor scanning behavior	*n* = 24 Field loss 12 Controls 12	Stroke Chronic visual field defects	Compensatory: audio–visual stimulation of blind hemifield.	4 hr daily over a period of 2 weeks	Patients reported improvement in ocular exploration after audio‐visual training, leading to a reduction in total exploration time.
Plow et al. (2010) Article taken from cochrane review Pollock et al. (2012)	RCT	To test the effect of transcranial direct current stimulation to enhance VRT	*n* = 8	Stroke	Restitutive: VRT with active tDCS vs VRT with sham tDCS	VRT = 30 min 2× daily for 3 months Active tDCS = 2 mA/min along with VRT sham tDCS = 30 s ramped down to 0 then turned off, along with VRT	Results of preliminary case comparisons suggest that occipital cortical tDCS may enhance recovery of visual function associated with concurrent VRT through visual cortical reorganization.
Plow et al. (2012)	Double blinded RCT (pilot)	To investigate whether training eye movements induces change in the neural activity of cortical visual areas.	*n* = 12 (8 in final analysis)	Mixed Stroke *n* = 1 Surgical trauma *n* = 2 At least 3 months poststroke	Restitutive: VRT compared with active tDCS Control group received sham tDCS	30 min of training, 3× a week For 3 months	In 8 patients tested, the VRT and tDCS group demonstrated significantly greater expansion in visual field and improvement on ADL's.
Poggel et al. (2004) Article taken from cochrane review Pollock et al. (2012)	RCT	To determine whether attentional cueing improves VRT	*n* = 20	Mixed	Restitutive: VRT with attentional cueing vs VRT with no attentional cueing	30–35 min 2× daily, for 56 sessions For approx. 1 month	In the area of the cue, restoration of vision was significantly greater than during VRT without cueing.
Pollock et al. (2012) (Pollock et al., 2011)	Cochrane systematic review	To determine the effects of interventions for visual field defects after stroke	*n* = 344 13 studies	Mixed Stroke *n* = 285	Various Resistutive *n* = 5 Compensatory *n* = 5Substitutive *n* = 3	Various	There is limited evidence to support the use of scanning training. There is insufficient evidence for the benefit of VRT or prisms.
Rossi et al. (1990) Article taken from cochrane review Pollock et al. (2012)	RCT	To determine whether fresnel prisms improve visual perception	*n* = 30	Stroke	Substitutive: 15∆ hemi‐circular fresnel prisms applied to glasses along with standard rehabilitation	Worn all day for 4 weeks	After four weeks the prism treated group performed significantly better than the control group.
Roth et al. (2009) Article taken from cochrane review Pollock et al. (2012)	RCT	To compare explorative saccade and flicker training	*n* = 30	Mixed Stroke/Hemorrhage *n* = 26 Other *n* = 4	Compensatory: exploratory eye scanning training Restitutive: flicker‐stimulation training	Both groups = 30 min 2× daily, 5 days a week for 6 weeks	Explorative saccadic training selectively improves saccadic behaviou, natural search and scene exploration on the blind side.
Rowe, Conroy, et al. (2016)	Prospective three‐arm RCT	To compare prism therapy and visual search training for hemianopia to standard care (information only)	*n* = 87	Stroke Stable hemianopia	Compensatory: visual search training and advice Substitutive: prism therapy	Visual search: 30 min daily for minimum of 6 weeks Fresnel prisms: minimum 2 hr daily for a minimum of 6 weeks	Visual search training had significant improvement in vision‐related quality of life. Prism therapy produced adverse events in 69%.
Schmielau and Wong (2007)	Cohort study	To evaluate whether restoration of visual field in patients with hemianopia is possible using the Lubeck Reaction Perimeter (LRP)	*n* = 20	Mixed Infarction *n* = 11 Hemorrhage *n* = 7 Trauma *n* = 2	Restitutive: VRT using the LRP	45 min of training, 2× a week Average length of training 8.2 months	17 out of 20 patients showed a stable and significant increase in visual field size.
Schuett et al. (2012)	Randomized crossover design	To determine whether training‐related improvements in reading and visual exploration with compensatory therapies are task specific	*n* = 36	Mixed Stroke *n* = 34 Tumor *n* = 2	Compensatory: software‐based reading and visual exploration training	Group A: visual exploration training then reading training Group B: converse Both groups 45‐min sessions in 10 units (30 trials each)	Findings demonstrate tha the training related improvements in reading and visual exploration are highly specific and task dependent, and there was no effect of training sequence.
Spitzyna et al. (2007) Article taken from cochrane review Pollock et al. (2012)	RCT	To determine whether optokinetic therapy improves test reading for hemianopic dyslexia	*n* = 22	Mixed	Compensatory: optokinetic nystagmus inducing reading therapy	4 weeks of training (minimum of 400 min of rehabilitation) 20× 20 min sessions	OKN inducing therapy preferentially affects reading saccades in the direction of the induced (involuntary) saccadic component.
Szlyk et al. (2005) Article taken from cochrane review Pollock et al. (2012)	Randomized crossover design	To assess the use of prisms for navigation and driving for patients with hemanopia	*n* = 10	Mixed population Injury involving occipital lobe only	Sustitutive: Gottlieb visual field awareness system 18.5 dioptre lens vs 20 dioptre fresnel prisms	Training of 4× 2–3 hr indoor sessions with LVA specialist and 8× 2 hr outdoor sessions behind the wheel Prisms worn for 3 months	Patients with hemianopia showed improvements in visual functioning using prism lenses, although these improvements were smaller than those found in previous studies.
Taylor et al. (2011)	Quasi‐experimental feasibility study	To evaluate a systematic treatment program that targeted aspects of visual functioning affected by visual field deficits following stroke	*n* = 15	Stroke	Compensatory: Experimental Group—scanning therapy	Experimental group: 30 min sessions 2× weekly. For 4 weeks Conventional treatment group: usual OT therapy (various durations)	Introduction of the systematic treatment programme resulted in a significant change in scores of the Nottingham Adjustment scale.
Weinberg et al. (1977) Article taken from cochrane review Pollock et al. (2012)	RCT	To test the effect of visual scanning training on reading‐related tasks	*n* = 57	Stroke	Compensatory: visual scanning training	1 hr a day for 4 weeks Total: 20 hr of training	The training group showed superior results to the control group.

*Note*

Articles taken from Cochrane review (*n* = 9) included for information only and are not included in this adaptation review.

#### 3.2.1 Quality assessment

The quality of evidence was assessed for each of the 30 articles included in this section (Supporting Information Tables [Link], [Link], [Link], [Link]). In summary, two articles scored 100% for quality of evidence presentation in the opinion of the reviewers (Gall & Sabel, 2012; Ong et al., 2012). Twenty five articles scored between 75% and 99% and therefore deemed as good quality evidence. Three scored between 50% and 74% on the relevant quality checklists.

Interventions for visual field defects are proposed to work in multiple ways, as detailed by a Cochrane review of such interventions (Pollock et al., 2011). This Cochrane review investigated the effectiveness of visual field loss interventions in three intervention categories: restitution, compensation, and substitution. The primary outcome measure used for this review was functional ability in activities of daily living, with secondary outcome measures including extended activities of daily living, visual field, balance, falls, depression/anxiety, discharge destination, quality of life, visual scanning, adverse events, and death. The review was limited to randomized trials and studies included in Cochrane systematic reviews involving adult stroke patients, and a total of thirteen studies met the authors’ inclusion criteria (Roth et al., 2009; Bainbridge & Reding, 1994; Carter, Howard, & O'Neil, 1983; Jobke, Kasten, & Sabel, 2009; Kasten, Wüst, Behrens‐Baumann, & Sabel, 1998; Kasten, Bunzenthal, Müller‐Oehring, Mueller, & Sabel, 2007; Plow et al., 2010; Poggel, Kasten, & Sabel, 2004; Rossi, Kheyfets, & Reding, 1990; Spitzyna et al., 2007; Szlyk, Seiple, Stelmack, & McMahon, 2005; Weinberg et al., 1977, 1979). The Cochrane authors concluded there is some limited evidence to support the use of compensatory scanning therapy to improve scanning and reading outcomes in this patient group. At the time of review publication, there was not sufficient evidence to support the impact of this compensatory scanning therapy on functional activities undertaken by the stroke survivor. In addition, there was insufficient evidence to reach conclusions regarding the benefits of visual restitution training (VRT) or prisms for this cohort of patients.

#### 3.2.2 Compensatory treatment

The aim of compensatory treatments is to bridge the gap between a person's abilities and the demands of everyday tasks. In other words, to aid a person's ability to compensate or adapt for the visual impairment they are experiencing. Compensatory therapies involve improving a persons’ visual search or scanning techniques and may include paper‐based tasks and/or computer training programs. Hazelton et al. explored the effect and feasibility of home‐based scanning techniques for rehabilitation by comparing four intervention types: paper‐based rainbow readers, computer software VISIOcoach, web‐based Happy Neuron, and specialized NeuroVision training. In this small sample study, they concluded that home‐based training is a feasible option and that the key factors for maximizing intervention potential include levels of cognitive impairment and participant perceptions. Free web‐based therapies are widely available in the form of Eye‐search (http://www.eyesearch.ucl.ac.uk) and Read‐right (http://www.readright.ucl.ac.uk), and their development has improved access to compensatory therapies for stroke survivors with visual field loss (Ong et al., 2012, 2015).

There have been some favorable outcomes demonstrated with audio–visual stimulation of the visual field (Bolognini, Rasi, Coccia, & Làdavas, 2005; Keller & Lefin‐Rank, 2010; Passamonti, Bertini, & Làdavas, 2009). This is a developing area of compensatory therapy which involves the use of acoustic as well as visual stimuli during the training process and has the potential for further development of effective techniques in compensatory rehabilitation. A review of the literature by Tinga et al. (2016) attempted to explore the evidence base for multisensory stimulation as a possible rehabilitation method for functional recovery in patients with sensory deficits after stroke. The review focuses on recovery and not adaptation so was not included in this systematic review; however, the authors highlight a lack of evidence in this field of research. A valuable next step would be to investigate the effect of multisensory stimulation with well‐designed randomized control trials, to explore the effects on visual field loss, in both the areas of recovery and adaptation.

The majority of studies relating to compensatory treatments are concerned with the improvement of eye movements and scanning into the affected field (Bergsma et al., 2011; Roth et al., 2009; Aimola et al., 2014; Hazelton, Pollock, Walsh, & Brady, 2015; Jacquin‐Courtois, Bays, Salemme, Leff, & Husain, 2013; Lane, Smith, Ellison, & Schenk, 2010; Pambakian, Mannan, Hodgson, & Kennard, 2004; Kerkhoff, Münssinger, & Meier, 1994; Mazer et al., 2003; Nelles et al., 2010; Taylor, Poland, Harrison, & Stephenson, 2011; Schuett, Heywood, Kentridge, Dauner, & Zihl, 2012), as well as increased saccadic movements into the affected field (Mannan, Pambakian, & Kennard, 2010; Lévy‐Bencheton et al., 2016; Kerkhoff, Münßinger, Eberle‐strauss, & Stögerer, 1992). A number of studies have specifically reported on subjective improvements in activities of daily living following compensatory therapy, such as improvements in mobility, reading, driving, and detection of obstacles (Bergsma et al., 2011; Ong et al., 2015; Keller & Lefin‐Rank, 2010; Aimola et al., 2014; Jacquin‐Courtois et al., 2013; Kerkhoff et al., 1994; Mazer et al., 2003; de Haan, Melis‐Dankers, Brouwer, Tucha, & Heutink, 2015; Hayes, Chen, Clarke, & Thompson, 2012; Nelles et al., 2001; Rowe, Conroy, et al., 2016). A study by de Haan et al. (2015) examined the effect of compensatory scanning training on mobility‐related activities and found a link between visual scanning training and detection of peripheral stimuli and obstacle avoidance. This evidence provides further support for the role of compensatory treatment in the adaptation process.

A recently published pilot randomized controlled trial compared the effectiveness of visual search compensatory training to standard care and the substitutive treatment of prism therapy (Rowe, Conroy, et al., 2016). Results from this trial showed significant improvements in vision‐related quality of life measures for participants undergoing visual search training, highlighting the need for further research in this area.

#### 3.2.3 Substitutive treatment

Substitution interventions involve adaptation to visual field loss using optic devices, mechanical aids, or modifications to the immediate environment. Studies included in this review concerning substitutive treatments describe the use of prisms for hemianopia (Bowers, Keeney, & Peli, 2014; Giorgi, Woods, & Peli, 2009). The interventions Cochrane review (Pollock et al., 2011) reported insufficient evidence to reach any generalized conclusions regarding the effectiveness of substitutive interventions (prisms) compared to a placebo, control, or no treatment. A study by Giorgi et al. (2009) evaluated the use of peripheral prism glasses in an extended wearing trial. They describe a reported benefit in patients completing the study, with 42% choosing to continue to wear the prisms at long‐term follow‐up. However, there was no significant difference in perceived quality of life questionnaire scores (NEI‐VFQ‐25) between weeks one and six of prism wear. A later randomized crossover trial by Bowers et al. (2014) investigated real peripheral prisms (57 prism dioptre) in comparison with sham prisms (five prism dioptres) as a treatment method for homonymous hemianopia. Results showed that the difference between the proportion of participants preferring real to sham prisms at the end of the first crossover period was not significant, but was significant at the end of the second period. In total, 61% continued prism wear with an equal number from the oblique and horizontal position groups.

Rowe, Conroy, et al. (2016) report a pilot randomized controlled trial comparing the effectiveness of visual search compensatory training to standard care and the substitutive treatment of prism therapy. In this trial, eighteen patients (69%) in the Fresnel prism treatment arm experienced a total of 42 adverse events including headaches, diplopia, and visual confusion, versus 7% of patients in the visual search arm reporting adverse events (fatigue). Participants in the visual search arm continued treatment after the trial treatment period in greater numbers than participants in the Fresnel prism group; 24 versus 14 participants after 6 weeks, 21 versus 12 after 12 weeks, and 10 versus 5 after 26 weeks, respectively.

#### 3.2.4 Restitution treatment

Restitutive interventions include those where there is direct training or repetitive stimulation of the impaired visual field (Pollock et al., 2011). Visual restoration therapy (VRT) is one form of restitution treatment that is the most commonly reported in the literature. The aim of VRT is the improvement of visual field loss by stimulating the border along the area of visual field loss; along the boundary between remaining, normal visual field and damaged, impaired visual field. Pollock et al. (2011) conclude that there is insufficient evidence to draw conclusions about the effectiveness of VRT as compared to placebo, control, or no treatment when focused on visual field outcomes. This is further supported by Roth et al. (2009) and Reinhard et al. (2005) who examined whether VRT has the potential to change absolute hemianopic field defects, reporting none of their seventeen patients to have an explicit change in defect after training. The latter study was not included in the review as its focus was on recovery of visual field following VRT and not adaptation. In trials where eye movement recording was not undertaken, improvement in visual field due to eye movements cannot be excluded (Reinhard et al., 2005; Schmielau & Wong, 2007). However, studies where eye movements were measured did confirm visual field recovery, arguing against the hypothesis that compensatory eye movements alone can explain vision recovery (Gall et al., 2016; Kasten, Bunzenthal, & Sabel, 2006).

A number of studies do report variable expansion of the visual field following VRT treatment (Gall & Sabel, 2012; Schmielau & Wong, 2007; Marshall, Chmayssani, O'Brien, Handy, & Greenstein, 2010; Plow, Obretenova, Fregni, Pascual‐Leone, & Merabet, 2012). There is significant variation in the treatment dose, duration, and field outcome for these studies.

Although the aim of VRT is restitution and not adaptation specifically, the practices of VRT are reported to affect quality of life measures (Gall & Sabel, 2012), hence, having potential to influence the adaptation process. The effect of VRT on the absolute visual field defect is outside the aims and objectives of this review.

## 4 CONCLUSION

There is substantial evidence that patients can be supported to compensate and adapt to visual field loss following stroke using a range of strategies and methods. However, this systematic review highlights the fact that many unanswered questions remain: what does adaptation to visual field loss mean to the patient, carer, and clinician? How can adaptation be measured over time? Why do some people adapt more effectively and at a quicker rate than others, despite seemingly similar rehabilitation opportunities and experiences? If these questions can be answered through high quality observations and assessments then this would be a valuable starting point for understanding adaptation. Until we can understand these processes and what factors are important, targeted interventions may have a limited effect. This systematic review is the starting point for a clinical study exploring the factors that are important for the adaptation to poststroke visual field loss, taking into consideration a multitude of factors such as age, site of stroke, extent of visual field loss, previous scanning experiences, and rehabilitation scanning treatment.

It is important to note that some studies in this review observed a mixed caseload and therefore did not focus on a specific stroke survivor population. However, in the authors’ opinion, the cause of visual field defect is not likely to be a crucial factor for the adaptation process, but indeed a range of other factors will show a greater influence. Future research should consider the factors that could be important for the adaptation process, seeking views of stroke survivors themselves and their families/carers to identify aspects they feel are important for their own adaptation journey, as well as clinicians responsible for the rehabilitation of this population group.

As clinicians working with this group of patients, we are expected to make a clinical judgment on whether a person has adapted to their loss of peripheral vision. This is particularly true for a situation where a person wants to consider a return to driving with a hemianopia under the exceptional cases rule for visual field loss. One of the Driving and Vehicle Licensing Agency (DVLA) (DVLA, 2018) requirements for consideration for the exceptional cases ruling to return to driving despite having a significant visual field loss is “clinical confirmation of full functional adaptation” to the visual field loss. There is currently no guidance on what this actually means or how clinicians can test for this, creating inconsistent approaches for patients and inconsistent care and decision making regarding referral of patients for driving assessment. This is an area that must be addressed in the interest of equality for those with visual impairment.

It is vital that the factors important for adaptation be identified to allow clinicians to recognize which people are likely to have difficulty adapting and target interventions specifically within these areas, as well as to develop methods for assessing adaptation and monitoring change over time.

## CONFLICT OF INTEREST

The authors report no conflicts in relation to this review. Claire Howard and Fiona Rowe are funded by National Institute for Health Research Fellowships to carry out research, which includes this systematic review. The views expressed are those of the authors and not necessarily those of the NHS, the NIHR, or the Department of Health.
