# Supplementary material for: Adaptation to poststroke visual field loss: A systematic review
Source: Brain Behav. 2018 Jul 13;8(8):e01041. doi: 10.1002/brb3.1041 (PMC6086007; doi:10.1002/brb3.1041)
Supplement: Supplementary file 1 [file BRB3-8-e01041-s001.docx]

**Table S1: Quality assessment of adaptation papers using the CONSORT checklist**

|  |  |  | Loverro et al 1988 |
| --- | --- | --- | --- |
| Introduction | Objectives | 2b | + |
| Methods | Trial design | 3a | - |
|  | Changes to methods | 3b | - |
|  | Eligibility | 4a | + |
|  | Interventions for each group | 5 | - |
|  | Outcome measures | 6a | + |
|  | Changes to trial outcomes | 6b | - |
|  | Sample size | 7a | - |
|  | Interim analysis | 7b | n/a |
|  | Method of random allocation sequence | 8a | + |
|  | Randomisation | 8b | - |
|  | Implementation of random allocation | 9 | - |
|  | Generation of random allocation | 10 | + |
|  | Blinding | 11a | - |
|  | Similarity of interventions | 11b | + |
|  | Statistical methods | 12a | + |
|  | Additional analyses | 12b | + |
| Results | No. of participants | 13a | + |
|  | Losses and exclusions | 13b | + |
|  | Dates of recruitment - follow up | 14a | - |
|  | Reason trial ended | 14b | - |
|  | Baseline demographic | 15 | + |
|  | Analysis of original assigned groups | 16 | + |
|  | Results with precision | 17a | + |
|  | Additional analysis | 18 | + |
|  | Harms | 19 | - |
| Discussion | Limitations | 20 | - |
|  | Generalisability | 21 | - |
|  | Consistent interpretation | 22 | - |
| Other Info | Access to full protocol | 24 | - |
| Overall % |  |  | 48 |

-

= Not reported = Unclear = Reported

+

?
