# Supplementary material for: Adaptation to poststroke visual field loss: A systematic review
Source: Brain Behav. 2018 Jul 13;8(8):e01041. doi: 10.1002/brb3.1041 (PMC6086007; doi:10.1002/brb3.1041)
Supplement: Supplementary file 2 [file BRB3-8-e01041-s002.docx]

**Table S2: Quality assessment of adaptation papers using the STROBE checklist**

|  | Introduction | Methods | | | | | | | | Results | | | | | Discussion | | | | Overall percentage (%) |
| --- | --- | --- | --- | --- | --- | --- | --- | --- | --- | --- | --- | --- | --- | --- | --- | --- | --- | --- | --- |
|  | 3 | 4 | 6 | 7 | 8 | 9 | 10 | 11 | 12 | 13 | 14 | 15 | 16 | 17 | 18 | 19 | 20 | 21 |  |
| Cassidy et al 1999 | + | + | + | + | + | + | - | + | + | + | + | + | + | n/a | + | - | + | + | 88 |
| Hardiess et al 2010 | + | + | + | + | + | + | - | + | + | + | + | + | + | n/a | + | + | + | + | 94 |
| Kasneci et al 2014 | + | + | + | + | + | - | + | + | + | + | + | + | + | n/a | + | + | + | + | 94 |
| Machner et al 2009 | + | + | - | + | + | + | - | + | + | + | + | + | + | n/a | + | - | + | + | 82 |
| McDonald et al 2005 | + | + | ? | + | + | + | - | + | + | + | + | + | + | n/a | + | + | + | + | 88 |
| Pambakian et al 2000 | + | + | + | + | + | - | - | + | + | + | + | + | + | n/a | + | + | + | + | 88 |
| Papageorgiou et al 2012 | + | + | + | + | + | - | - | + | + | + | + | + | + | n/a | + | - | + | + | 82 |
| Rowe and VIS Group 2013 | + | + | + | + | + | n/a | + | + | + | + | + | + | + | n/a | + | - | + | + | 93 |
| Tant et al 2002 | + | + | + | + | + | n/a | - | + | + | + | + | + | + | n/a | + | - | + | + | 88 |
| Wood et el 2011 | + | + | + | + | + | + | - | + | + | + | + | + | + | + | + | + | + | + | 94 |
| Zangemeister and Oechsner 1996 | + | + | + | + | + | - | - | + | + | + | + | + | + | n/a | + | + | + | + | 88 |
