# Supplementary material for: Adaptation to poststroke visual field loss: A systematic review
Source: Brain Behav. 2018 Jul 13;8(8):e01041. doi: 10.1002/brb3.1041 (PMC6086007; doi:10.1002/brb3.1041)
Supplement: Supplementary file 4 [file BRB3-8-e01041-s004.docx]

**Table S4: Quality assessment of intervention papers using the CONSORT checklist**

|  | Introduction | Methods | | | | | | | | | | | | | | | | Results | | | | | | | | | | Discussion | | | Other info |
| --- | --- | --- | --- | --- | --- | --- | --- | --- | --- | --- | --- | --- | --- | --- | --- | --- | --- | --- | --- | --- | --- | --- | --- | --- | --- | --- | --- | --- | --- | --- | --- |
|  | Objectives | Trial design | Changes to methods | Eligibility | Interventions | Outcome measures | Changes to outcomes | Sample size | Interim analysis | Method of random allocation | Randomisation | Implementation of random | Generation of random | Blinding | Similarity of interventions | Statistical methods | Additional analyses | No. of participants | Losses and exclusions | Dates of recruitment – fu | Reason trial ended | Baseline demographics | Analysis of original assigned | Results with precision | Binary Outcomes | Additional analysis | Harms | Limitations | Generalisability | Consistent interpretation | Access to full protocol |
|  | 2b | 3a | 3b | 4a | 5 | 6a | 6b | 7a | 7b | 8a | 8b | 9 | 10 | 11a | 11b | 12a | 12b | 13a | 13b | 14a | 14b | 15 | 16 | 17a | 17b | 18 | 19 | 20 | 21 | 22 | 24 |
| Aimola et al 2014 | + | + | - | + | + | + | + | + | n/a | + | + | + | - | + | n/a | + | + | + | + | + | - | + | + | + | n/a | + | - | + | + | + | + |
| Bowers et al 2014 | + | + | - | + | + | + | - | + | n/a | + | + | + | + | + | + | + | + | + | + | + | - | - | + | + | + | + | - | + | + | + | + |
| de Haan et al 2015 | + | + | - | + | + | + | n/a | + | n/a | + | + | + | + | + | + | + | + | + | + | + | - | + | + | + | + | + | + | + | + | + | + |
| Keller & Lefin-Rank 2010 | + | + | - | + | + | + | n/a | - | n/a | + | + | + | - | + | + | + | + | + | + | + | - | + | + | + | n/a | + | - | + | + | + | - |
| Mazer et al 2003 | + | + | + | + | + | + | n/a | + | + | + | + | - | ? | + | + | + | + | + | + | + | - | - | + | + | + | + | + | + | + | + | + |
| Plow et al 2012 | + | + | - | + | + | + | - | + | n/a | - | - | - | - | + | + | + | + | + | + | ? | - | + | + | + | + | + | + | + | - | + | - |
| Rowe et al 2016 | + | + | - | + | + | + | n/a | + | n/a | + | + | + | + | + | + | + | + | + | + | + | + | + | + | + | + | + | + | + | + | + | + |
| Schuett et al 2011 | + | + | - | + | + | + | n/a | - | n/a | + | - | - | + | - | + | + | + | + | + | + | - | + | + | + | - | + | + | + | + | + | - |

-

= Not reported = Unclear = Reported

+

?
