# Supplementary material for: Adaptation to poststroke visual field loss: A systematic review
Source: Brain Behav. 2018 Jul 13;8(8):e01041. doi: 10.1002/brb3.1041 (PMC6086007; doi:10.1002/brb3.1041)
Supplement: Supplementary file 5 [file BRB3-8-e01041-s005.docx]

**Table S5: Quality assessment of intervention papers using the STROBE checklist**

|  | Introduction | Methods | | | | | | | | Results | | | | | Discussion | | | | Overall percentage (%) |
| --- | --- | --- | --- | --- | --- | --- | --- | --- | --- | --- | --- | --- | --- | --- | --- | --- | --- | --- | --- |
|  | 3 | 4 | 6 | 7 | 8 | 9 | 10 | 11 | 12 | 13 | 14 | 15 | 16 | 17 | 18 | 19 | 20 | 21 |  |
| Bergsma et al 2011 | + | + | + | + | + | + | - | + | + | - | + | + | + | n/a | + | + | + | + | 88 |
| Bolognini et al 2005 | + | + | + | + | + | - | - | + | + | + | + | + | + | n/a | + | - | + | + | 82 |
| Gall and Sabel 2012 | + | + | + | + | + | + | + | + | + | + | + | + | + | n/a | + | + | + | + | 100 |
| Giorgi et al 2009 | + | + | + | + | + | + | + | + | + | + | + | + | + | - | + | - | - | - | 78 |
| Hayes et al 2012 | + | + | + | + | + | + | - | - | - | + | + | + | n/a | n/a | + | + | + | + | 81 |
| Hazelton et al 2015 | + | + | + | + | - | - | - | + | + | + | - | + | n/a | n/a | + | - | + | + | 69 |
| Kerkhoff et al 1992 | + | + | + | + | + | - | - | + | + | + | - | + | + | n/a | + | + | + | + | 82 |
| Kerkhoff et al 1994 | + | + | + | + | + | n/a | - | + | + | + | - | + | + | n/a | + | + | + | + | 88 |
| Lane et al 2010 | + | + | + | + | + | + | - | + | + | + | + | + | + | + | + | - | + | + | 89 |
| Levy-Bencheton et al 2016 | + | + | + | + | + | + | - | + | + | + | + | + | + | n/a | + | - | + | + | 88 |
| Mannan et al 2010 | + | + | + | + | + | + | - | + | + | + | + | + | + | n/a | + | - | + | + | 88 |
| Marshall et al. 2010 | + | + | + | + | + | + | - | + | + | + | + | + | - | n/a | + | + | + | + | 88 |
| Nelles et al 2010 | + | + | + | + | + | + | - | + | + | - | + | + | - | n/a | + | - | + | + | 76 |
| Ong et al 2012 | + | + | + | + | + | + | + | + | + | + | + | + | + | n/a | + | + | + | + | 100 |
| Ong et al 2015 | + | + | + | + | + | - | + | + | + | + | + | + | + | n/a | + | + | + | + | 94 |
| Pambakian et al 2004 | + | + | + | + | + | - | + | - | + | + | + | + | + | n/a | + | - | + | + | 82 |
| Passamonti et al 2008 | + | + | + | + | + | - | - | + | + | + | + | + | + | n/a | + | - | + | + | 82 |
| Schmielau & Wong 2007 | + | + | + | + | + | + | + | + | + | + | + | + | + | + | + | - | + | + | 94 |
| Taylor et al 2011 | + | + | + | + | + | + | - | + | + | + | + | + | + | n/a | + | + | + | + | 94 |
