# Supplementary material for: Adaptation to poststroke visual field loss: A systematic review
Source: Brain Behav. 2018 Jul 13;8(8):e01041. doi: 10.1002/brb3.1041 (PMC6086007; doi:10.1002/brb3.1041)
Supplement: Supplementary file 6 [file BRB3-8-e01041-s006.docx]

**Table S6: Quality assessment of intervention papers using the GRACE checklist**

|  | Data | | | | | | Methods | | | | | Overall percentage (%) |
| --- | --- | --- | --- | --- | --- | --- | --- | --- | --- | --- | --- | --- |
|  | Treatment | Primary Outcome | Primary Clinical Outcome | Validation | Outcome | Both groups measured equally | Population restriction | Comparison groups | Confounding variables | Immortal-time bias | Analyses |  |
|  | D1 | D2 | D3 | D4 | D5 | D6 | M1 | M2 | M3 | M4 | M5 |  |
| Jacquin-Courtois et al 2012 | + | + | + | + | + | + | - | - | + | + | + | 82 |
| Nelles et al 2001 | + | + | + | + | + | + | ? | - | + | + | + | 82 |
