# Supplementary material for: Adaptation to poststroke visual field loss: A systematic review
Source: Brain Behav. 2018 Jul 13;8(8):e01041. doi: 10.1002/brb3.1041 (PMC6086007; doi:10.1002/brb3.1041)
Supplement: Supplementary file 7 [file BRB3-8-e01041-s007.docx]

**Table S7: Quality assessment of intervention papers using the PRISMA checklist**

|  | Title | Abstract | Introduction | | Methods | | | | | | | | | | | | Results | | | | | | | | | | Funding |
| --- | --- | --- | --- | --- | --- | --- | --- | --- | --- | --- | --- | --- | --- | --- | --- | --- | --- | --- | --- | --- | --- | --- | --- | --- | --- | --- | --- |
|  | 1 | 2 | 3 | 4 | 5 | 6 | 7 | 8 | 9 | 10 | 11 | 12 | 13 | 14 | 15 | 16 | 17 | 18 | 19 | 20 | 21 | 22 | 23 | 24 | 25 | 26 | 27 |
| Pollock et al 2011 | + | + | + | + | - | + | + | + | + | + | + | + | + | + | + | + | + | + | + | + | n/a | + | n/a | + | + | + | - |
